# Effects of Facilitation vs. Exhibit Labels on Caregiver-Child Interactions at a Museum Exhibit

**DOI:** 10.3389/fpsyg.2021.637067

**Published:** 2021-03-12

**Authors:** Susan M. Letourneau, Robin Meisner, David M. Sobel

**Affiliations:** ^1^New York Hall of Science, New York, NY, United States; ^2^Boston Children's Museum, Boston, MA, United States; ^3^Brown University, Providence, RI, United States

**Keywords:** caregiver-child interaction, children's museums, facilitation, exhibit labels, exploration, informal learning

## Abstract

In museum settings, caregivers support children's learning as they explore and interact with exhibits. Museums have developed exhibit design and facilitation strategies for promoting families' exploration and inquiry, but these strategies have rarely been contrasted. The goal of the current study was to investigate how prompts offered through staff facilitation vs. labels printed on exhibit components affected how family groups explored a circuit blocks exhibit, particularly whether children set and worked toward their own goals, and how caregivers were involved in children's play. We compared whether children, their caregivers, or both set goals as they played together, and the actions they each took to connect the circuits. We found little difference in how families set goals between the two conditions, but did find significant differences in caregivers' actions, with caregivers in the facilitation condition making fewer actions to connect circuits while using the exhibit, compared to caregivers in the exhibit labels condition. The findings suggest that facilitated and written prompts shape the quality of caregiver-child interactions in distinct ways.

## Introduction

Decades of research on informal STEM learning has advocated for involving learners in actively exploring materials, solving problems, and making discoveries, rather than passively receiving information (see National Research Council, 2009; National Academies of Sciences, Engineering, and Medicine, 2018; for reviews). Active learning experiences allow children to use their direct interactions with the world to construct conceptual understandings and make connections to own their interests and prior knowledge (Zimmerman, 2007; Kuhn, 2011; Miller et al., 2018). Understanding how such learning takes place has informed shifts in curricula and pedagogical approaches toward inquiry- and project-based methods that frame science as a practice and engage learners in asking questions and seeking out answers (Lehrer and Schauble, 2007; Krajcik et al., 2008; National Research Council, 2012, 2013). As interactive learning environments, science centers and children's museums are designed to invite active exploration, and museums have developed well-tested strategies for designing exhibits that promote exploration and inquiry (Gutwill and Allen, 2010; Humphrey and Gutwill, 2017).

Children's interactions with their caregivers are a critical part of this learning process. Research on informal learning in general has articulated how children's conversations and everyday interactions with family members shape their learning across a wide range of settings (Rogoff et al., 2016). Within science centers and children's museums, family groups learn through their exploration of museum exhibits in the larger context of their social interactions, cultures, and everyday lives (Ellenbogen et al., 2004; Gutwill and Allen, 2010; Ash et al., 2012; Falk and Dierking, 2018). Caregivers support children's learning in many ways in these settings—by guiding children's attention or exploration, asking questions, offering explanations, and making connections to children's interests and prior experiences (e.g., Callanan and Jipson, 2001; Crowley et al., 2001; Fender and Crowley, 2007; Haden, 2010; Zimmerman et al., 2010).

Recognizing the value of caregiver-child interactions for children's learning and engagement, museum professionals have developed and refined different strategies for supporting families' interactions, particularly their shared exploration and inquiry. For example, museum exhibits can be designed to prompt active and sustained engagement by encouraging social interactions among members of a group or by requiring multiple people to work together (Humphrey and Gutwill, 2017). Likewise, facilitators in museums guide families' exploration of museum interactives by prompting conversations and encouraging deeper exploration of scientific concepts and phenomena (Piscitelli and Weier, 2002; Tran, 2008; King, 2009; Gutwill et al., 2015). Studies of caregiver-child interactions in museums also suggest that museums can support children's learning by prompting caregivers to use open-ended questions (Haden et al., 2014), by asking caregivers to encourage children's exploration or explanations (Van Schijndel et al., 2010; Willard et al., 2019), by instructing families about relevant scientific principles (Marcus et al., 2018), or by scaffolding families' scientific practices or inquiry behaviors (Gutwill and Allen, 2010).

Although informal learning research has largely focused on the benefits of family interactions, debates continue within the field of education about how much and in what ways adults should guide children's learning (Russ and Berland, 2019). Studies showing the value of caregiver-child interactions in museum settings exist alongside research in cognitive development demonstrating that adult involvement can sometimes limit children's curiosity and exploration. For example, seeing an adult demonstrate how to use a new toy can limit children's own exploration of it (Bonawitz et al., 2011), and children come to different causal conclusions when they make discoveries through their own actions than by watching the same actions performed by someone else (Kushnir and Gopnik, 2005; Sobel and Sommerville, 2010). Research on guided play responds to this tension by arguing that adults should offer guidance in open-ended ways while being attentive to children's own goals and interests (Weisberg et al., 2016; Baroody et al., 2019), and experimental studies tend to support this conclusion (Benjamin et al., 2010; Alfieri et al., 2011; Fisher et al., 2013; Haden et al., 2014).

In museum settings, caregivers interact with their children in many different ways, depending on their motivations for visiting, children's needs, prior knowledge, and cultural backgrounds (Swartz and Crowley, 2004; Gaskins, 2008; Beaumont, 2010; Downey et al., 2010; Fung and Callanan, 2013). Caregivers sometimes prefer to observe while children play, rather than being directly involved, focusing on the ways that children learn by interacting with museum exhibits and with other children (Wood and Wolf, 2010; Letourneau et al., 2017; Luke et al., 2019). Yet, many museums assume that caregivers' involvement in children's play is universally beneficial (Gaskins, 2008), when in fact the research paints a much more complex picture. For example, Medina and Sobel (2020) examined how caregivers and children explored a toy with causal functions, and found that when caregivers and children set goals together, children were more engaged and explored for a longer period of time than children whose caregivers were directive or who let children set their own goals. This work points to the need for more nuance in examining how caregivers' involvement affects children's engagement and learning in informal settings, and how museum practices might affect both the amount and the quality of caregiver-child interactions.

The current study builds on a line of collaborative research conducted in partnership with children's museums that examined caregiver-child interactions at museum exhibits. In one study across three children's museum sites, Callanan et al. (2020) examined children's exploration and caregiver-child explanations as they explored museum exhibits involving sets of gears. This study showed that caregivers' explanations prompted children to spin gears to test their causal properties, but children's causal thinking and persistence in solving problems (i.e., troubleshooting with the gears) was less affected by caregivers' involvement.

In a subsequent study, Sobel et al. (2020) examined whether and how caregiver-child interactions at a circuit block exhibit influenced children's engagement and learning when solving problems on their own with the same exhibit materials. The researchers recorded caregiver-child interactions as families played with a set of circuit blocks. They then asked children to complete a sequence of eight circuit challenges that increased in difficulty. They coded caregiver-child interactions using the same coding scheme as Medina and Sobel (2020), as well as the number of actions caregivers and children made in the 30 s before and the 30 s after completing common circuits, and the number of circuit challenges that children chose to attempt and completed on their own. Results suggested that children's engagement with the challenges was related to caregivers' involvement. Children in caregiver-directed dyads were subsequently less engaged in attempting to solve the challenges than children in child-directed or jointly-directed groups. Moreover, the more actions caregivers engaged in immediately before families completed circuits while playing together, the less able children were to construct those same circuits on their own later. Both of these findings suggest that children's autonomy in setting and completing goals is an important factor in their engagement and learning with this exhibit.

The current research extends this work to focus on the implications for practice—how might the design or facilitation of exhibits affect children's interactions with their caregivers? Specifically, this study aims to address two issues that remain relatively unexplored in research on caregiver-child interactions. The first is how the kinds of open-ended facilitation strategies that are commonly used in museums affect families' interactions. The existing research on facilitation in museum settings has either involved qualitative investigations of the wide range of practices used by facilitators to engage visitors (e.g., Tran, 2008; Gutwill et al., 2015), or experimental investigations of the impact of instructions from museum staff on how caregivers facilitate children's exploration of an exhibit (e.g., Gutwill and Allen, 2010; Van Schijndel et al., 2010; Haden et al., 2014). For example, most studies involve giving caregivers information before families begin exploring exhibits, in the form of written or verbal instructions about how to guide conversations with their children about exhibits (e.g., Benjamin et al., 2010; Haden et al., 2014; Willard et al., 2019), how to support children's exploration (Van Schijndel et al., 2010), or instructions about relevant scientific principles (Benjamin et al., 2010; Haden et al., 2014; Marcus et al., 2017). These types of structured interventions, however, are rarely used by facilitation staff in museums, although similar types of information may be available to families in the form of labels, signage, or multimedia displays. More commonly, facilitators in children's museums and science center's tend to offer brief and open-ended prompts to support and extend families' exploration and conversation throughout their interaction with exhibits.

The second unexplored issue is the relative benefits of facilitation as compared to physical design strategies like exhibit labels for supporting family interactions. Studies in museums have generally contrasted families' exploration of facilitated exhibits with their exploration of the same exhibits without facilitators present. In situations without facilitators, however, museums generally rely on the design features of the exhibit (such as labels or images) to convey information or prompt visitors' exploration.

These gaps in the research are significant because observational studies of facilitators' interactions with families in museum exhibits suggest that facilitators' presence can sometimes limit interactions between caregivers and their children (Pattison et al., 2018), and that caregivers may disengage or reject the assistance of facilitators when their involvement is seen as intrusive or overly didactic (Marino and Koke, 2003; Pattison and Dierking, 2013). More research is needed to examine how facilitation strategies commonly used in museum settings influence caregiver-child interactions in order to gain a more complete understanding of the roles that facilitators can play in supporting children's learning at museum exhibits.

**Figure 1:**
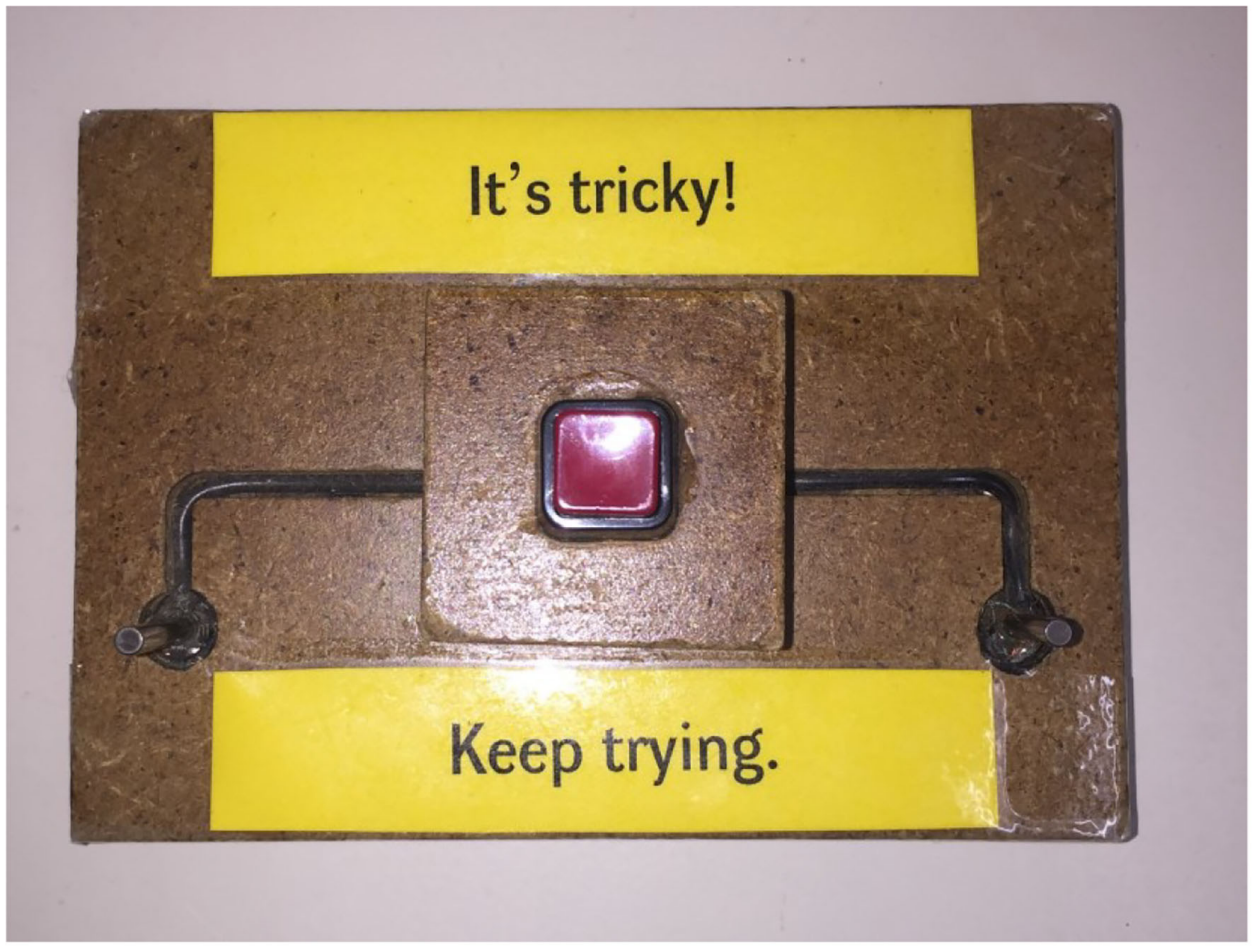
Example circuit block used in Exhibit label condition. See Table 1 for all block/label pairs.

To address these issues, we used the same circuit block exhibit as in Sobel et al. (2020) to examine how prompts offered by a facilitator or by exhibit labels affect the goals that children (ages 4–7, the same age range used in our previous study) and caregivers set as they play together, and the actions they each take to make discoveries with the exhibit. We compared how families played at a circuit block exhibit in which the same set of prompts were offered either by a facilitator or by labels printed on the circuit blocks themselves, making it impossible for families to use the circuit blocks without reading these messages (see Figure 1 for an example). We based the prompts on the types of open-ended questions that museum practitioners typically asked as families explored the exhibit, and the prompts were generated with input from museum staff. Prompts included open-ended questions to encourage children and caregivers to try connecting the blocks in different ways (e.g., “What can you connect to this?,” “How many things can you connect?”), questions to prompt observations that might prompt further exploration (e.g., “How fast can it spin?”), suggestions about things to try with the blocks (e.g., “Can you make two things go at the same time?”) and general encouragement to keep exploring (e.g., “It's tricky. Keep trying!,” “What else can you try?”). This set of prompts allowed for a more naturalistic experimental intervention that was both informed by and directly relevant to pedagogical practices in children's museums.

We examined whether multiple aspects of caregiver-child interactions differed when families received facilitated vs. written prompts, including: (1) overall caregiver-child interaction style, which reflected whether children, caregivers, or both set goals throughout their entire exploration of the exhibit; (2) the number of goal statements caregivers and children made as they explored the exhibit; and (3) how active children and caregivers were in the moments leading up to completing a circuit (relative to the moments after circuits were completed). We focused on these aspects of caregiver-child interaction because our prior study (Sobel et al., 2020) showed that directive interaction styles were negatively associated with children's engagement, and more actions on the part of caregivers prior to connecting the circuits were negatively associated with children's learning. Therefore, in the current study, we wished to specifically examine whether and how facilitated vs. written prompts affected these same aspects of caregiver-child interactions. Additional analyses of other aspects of dyads' language (e.g., praise, causal connections) are included in the Supplementary Material section.

## Methods

### Participants

Our sample consisted of 95 children between the ages of 4 and 7 (*M*_*age*_ = 72.27 months, SD = 14.26 months, Range = 48.00–96.00 months, 46 girls and 49 boys) each tested with at least one caregiver. Families were recruited and tested at a local children's museum (Providence Children's Museum in Providence, RI, United States) during the families' museum visits. This sample did not include any children who had participated in our previous study with this exhibit (Sobel et al., 2020). This sample size was chosen based on a set of power analyses done in G^*^Power3.1.9, based on analyses between the two conditions and the three caregiver-child interaction styles (described below), assuming α = 0.05 and β = 0.20 with a medium effect size. These analyses suggested that we needed a sample size between 88 and 108 participants given the analyses we planned on conducting (see below). These data were collected between June-August, 2017. Our final sample size was determined based on the number of visits to the museum we were able to conduct.

### Demographics of the Sample

Children were tested with at least one legal guardian present (referred to here as “caregivers”). Thirty-eight children in this sample were tested with only an adult caregiver present. The remaining 57 children were tested with a caregiver as well as other family members. Seventy-nine children were tested with a female caregiver; 16 were tested with a male caregiver. Caregivers were asked to fill out a questionnaire to gather demographic information as part of the procedure (see *Procedure*, below); demographic data are summarized in Table 1. Caregivers were asked to describe their family's ethnicity and race, as well as languages spoken at home, by writing in open-ended responses. Responses about race were grouped based on the most frequently reported categories in our sample (e.g., caregivers who referred to themselves as “Chinese” were categorized as Asian/Asian American).

**Table 1:** Demographic information.

**Variable**	**Response category**	**Number of dyads**
Household income	Below 30 K	10
	30–50 K	14
	50–70 K	17
	70–90K	11
	90–120 K	16
	Above 120 K	23
	No response	4
Caregivers' education level	Some HS	1
	HS Diploma	10
	Some College/Associates	23
	BA	25
	MA (or equivalent)	26
	PhD (or equivalent)	7
	No response	3
# Museum visits in past year	First-time visitor	23
	1–2 visits	14
	3–5 visits	19
	6–9 times	27
	10 or more visits	6
	No response	6
Caregiver age	21–35	40
	36–49	49
	50–65	3
	Over 65	2
	No response	1
Family ethnicity	Hispanic	19
	Non-hispanic	67
Family race	Black/African American	7
	Asian/Asian American	3
	Native American	0
	White/Caucasian	65
	Mixed/multiple races	11
	No response	9

Seventy-three caregivers reported that their families spoke only English at home. Four caregivers reported that the primary language spoken in the home was not English (Spanish and Chinese were reported). Fifteen reported that multiple languages were spoken in the home—always English and another language (Spanish, Cantonese, Urdu, Cape Verdean, Dutch, and Portuguese were listed). Three caregivers did not provide this information. Three dyads communicated in Spanish while playing with the exhibit. These videos were transcribed and translated by a native speaker, and coding (described below) was done from those transcripts.

### Materials

We constructed two sets of circuit blocks based on the circuit block exhibit at Providence Children's Museum. This exhibit was created as part of a project at the Museum that focused on highlighting the ways that children learn through play and exploration. Each set consisted of eight blocks: two blocks with LED lights (which could light up in two different colors depending on how the blocks were connected), two blocks with motorized spirals, two battery blocks, and two button blocks. A set of blocks was present on the table at the start of the procedure.

**Figure 2:**
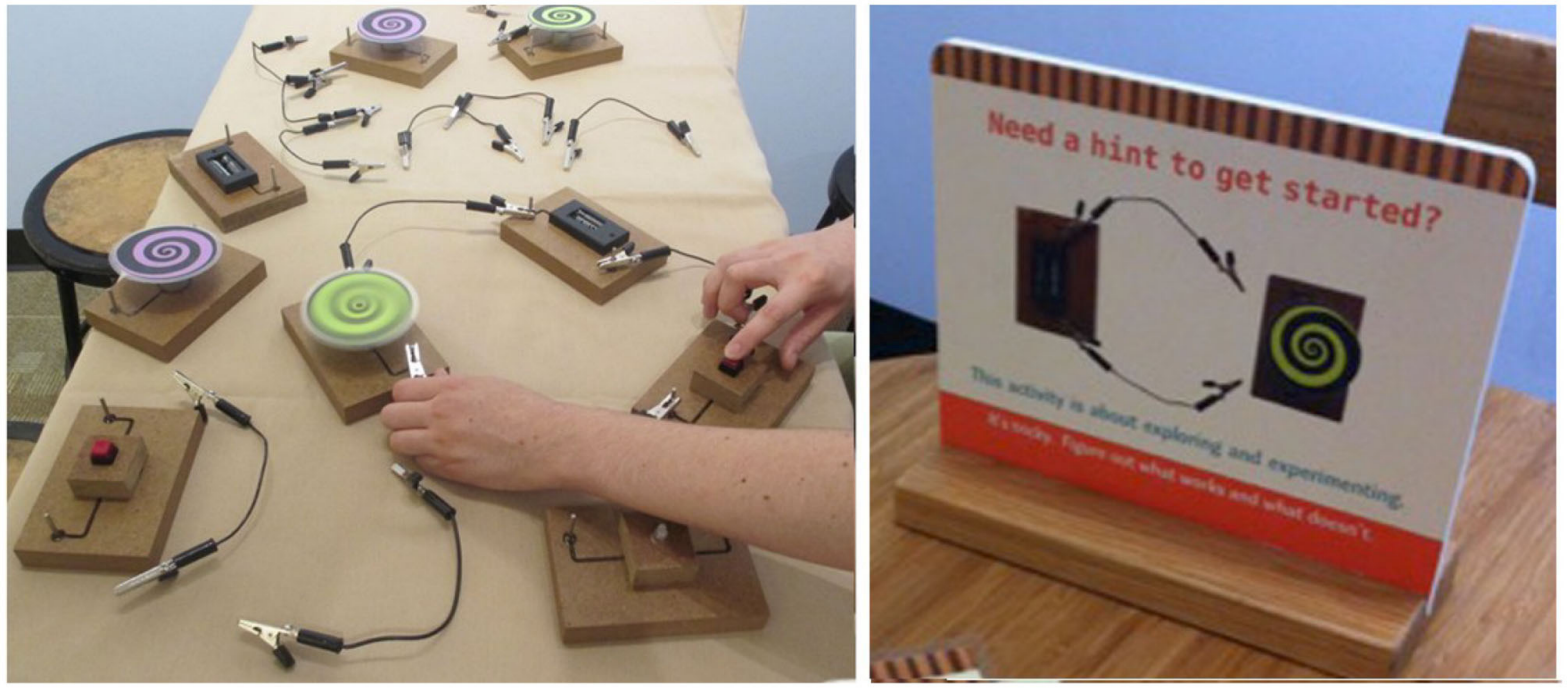
Picture of some of the circuit blocks and alligator clips in the facilitation condition (left), and hint sign present on the table in both conditions (right). The circuit blocks in the exhibit label condition were the same, but had yellow signs with black text on them, as in Figure 1. Note that not all circuit blocks used in the procedure are depicted. See text for details.

Also present on the table at the start of the procedure are a set of alligator clip wires (at least twenty) and a standing sign, which is normally part of the circuit block exhibit (see Figure 2). The sign shows a photo of a basic circuit with one battery block, one motor block, and two wires not fully connected, depicted from above, with a label reading, “Need a hint to get started? This activity is about exploring and experimenting. It's tricky. Figure out what works and what doesn't.” This image was meant to convey how to connect the circuit blocks, but not to give specific solutions or instructions in order to encourage open-ended exploration. The image appeared on both sides of the sign, and the caption appeared in English on one side and in Spanish on the other. In the facilitation condition, the blocks appeared as they do in Figure 2. The exhibit label condition used an identical set of blocks, except that each block had a label printed on it (printed only in English). All messages were in 20 pt. Arial font, printed on bright yellow paper. The labels are shown in Table 2.

**Table 2:** Labels printed on each block in the exhibit label condition.

**Block type**	**Label**
Battery	What can you connect to this?
Battery	What else can you try?
Button	Can you use this button to make something go?
Button	It's tricky! Keep trying.
Motor block with spiral	How fast can it spin?
Motor block with spiral	Can you make two things go at the same time?
LED	What color is the light?
LED	How many things can you connect?

### Procedure

The study procedures were approved under Brown University IRB protocol #1307000890, *Explaining, Exploring and Scientific Reasoning in Museum Settings*. Families were recruited at a local children's museum (Providence Children's Museum). If families agreed to participate, a researcher brought them into the room where the circuit block exhibit was located. The researcher was both a museum staff member and a member of the research lab, and therefore had familiarity with typical museum facilitation strategies with this exhibit.

After giving written consent and verbal assent when necessary^1^, families were asked to sit at the table with the exhibit materials, which included the eight circuit blocks, alligator clips, and hint sign. At the start of the procedure, one battery block and one effect block (either a spinner or a light) had an alligator clip attached to it, as an example of how the clips attached to the blocks. No circuits were completed at the start of the study and no two blocks were connected to one another at the start. This parallels the way in which the museum would typically set up the exhibit for regular use in between groups of visitors.

In both conditions, the researcher instructed groups to play with the circuit blocks however they liked, letting them know that they would have up to 15 min to play with the blocks. The researcher started the timer when the participating child approached the table. Groups were allowed to stop playing at any point they wished, but if they did not do so spontaneously, groups were given a 5-min and a 2-min warning before the 15-min was up. Families' interactions with the exhibit were recorded by a single video camera in the corner of the exhibit. The room was small enough that no additional microphones were needed to adequately capture families' conversations.

Because families visited the museum as a group, siblings and other members of the family group were also allowed to play with the circuits at the same time, but only one child per family participated in the study. A set of Squigz toys was available to entertain younger siblings when needed, while the participating child and at least one adult in the group played with the circuits.

Approximately half of the groups (*n* = 48) were randomly assigned to the *exhibit label condition*. In this condition, families were given the eight blocks with the labels on them, as depicted in Figure 1 and described in Table 2. The researcher introduced the activity as described above, and then waited outside the entrance to the room while families played with the circuits so as not to influence their behavior. The researcher only interacted with the dyads to give time limits or when the family indicated that they were finished playing.

The other half of the groups (*n* = 47) were assigned to the *facilitation* condition. In this condition, the blocks had no messages on them (as shown in Figure 2). After the researcher introduced the activity and allowed the family to sit at the table, she said “Can you make something go?” and then stepped back to allow families to begin playing, but remained nearby in the room throughout the entire time that the dyad played with the circuits.

In the facilitation condition, the researcher would stand next to the table where families were playing and offer prompts using the same language as what was written on the exhibits in the exhibit label condition whenever there was a pause in dyad's play with the circuits. For example, if the dyad paused after making a working circuit, the researcher would use one of the prompts in Table 2 to suggest a new action, possibly one that was slightly more complex than what the dyad just completed (e.g., if the dyad had just connected a motor to a battery, the researcher might use the prompts: “How fast can it spin?” or “Can you make two things go at the same time?”). Similarly, if children stopped playing or showed signs of frustration, the researcher would suggest a slightly easier activity, again using the same language as written on the blocks in the exhibit label condition (e.g., if the child paused without connecting any pieces, the researcher might use the prompts: “What can you connect to this?” [pointing to the battery block] or “What color is the light?”). The researcher kept track of the prompts that she offered, such that no prompts were repeated, and prompts were provided in only in English. If the caregiver stepped in to help, or if there was no pause in their play, the researcher would not intervene and would wait for a pause before offering another prompt. The researcher would continue offering prompts in this way throughout the entire duration of dyads' play with the exhibit.

The researcher did not provide other help or assistance, and was not involved in families' exploration of the circuit blocks except for observing and offering the verbal prompts described above. If families addressed her directly or asked her questions, she would give encouragement to keep trying (“Hm, I'm not sure! See if you can figure it out,” “It's tricky, but keep trying”) or vague observations or suggestions (e.g., “I wonder why that is,” “Maybe you can connect it a different way”) without giving away answers or giving direct instructions. Again, these messages were similar to what was written on some of the blocks in the exhibit label condition, and modeled after typical museum facilitation strategies with this exhibit. The goal with these neutral phrases was to allow the facilitator to be responsive to families, without offering praise, using leading questions, or giving any additional information that might influence their exploration.

Finally, if children showed a lot of frustration and wanted to stop, the researcher helped them connect the circuit they were attempting to complete before ending the study, so that children ended the study on a positive note. The majority of children, however, either played for the entire 15 min or indicated that they were ready to stop without showing signs of frustration. The prompts in the facilitation condition therefore varied slightly in their timing and order across the families who participated, in order to allow the researcher's interactions with families to be somewhat naturalistic, but the prompts families received included the same set of statements/questions that appeared in the exhibit label condition, and additional prompts offered by the facilitator in this condition did not contain any additional information about what to do with the circuit blocks or how to interact with them.

In both conditions, after families indicated that they were finished, caregivers were asked to complete a short demographic questionnaire, which included describing their experience visiting the museum, and the Attitudes toward Science questionnaire (Szechter and Carey, 2009), which measured their beliefs about the value of science and scientists. The results of this questionnaire are reported in the Supplementary Material section.

### Coding

We coded whether children or caregivers took the lead in setting goals for their exploration as they played together (based on overall interaction style and via the number of goal statements made by children and caregivers in their conversations, described below), as well as the number of actions taken by children and caregivers before and after groups completed circuits with the exhibit. We focused on these two coding categories based on the results of Sobel et al. (2020), who found that these measures independently related to children's engagement with or performance on a set of challenges with these exhibit materials. Our goal was to determine whether these aspects of caregiver-child interactions differed across the two conditions. Like Sobel et al., we also considered other facets of caregiver-child interaction, such as the language caregivers and children generated. This coding and analyses based on this coding are described in the Supplementary Material section because Sobel et al. (2020) found that it did not predict children's engagement with or learning at the exhibit.

#### Goal Setting

To measure goal setting, we examined two facets of caregiver-child interactions. First, we used a coding scheme based on work by Fung and Callanan (2013) and used by Sobel et al. (2020), which examined whether caregivers and/or children tended to set goals for their interaction. If multiple family members played with the exhibit, we considered only the actions of the caregiver and participating child in determining this code. Some dyads were *child-directed;* children both set goals and accomplished goals for themselves; these caregivers were passive during the interaction and allowed children to explore freely or simply offered encouragement. Some dyads were *caregiver*-*directed*; caregivers both set goals for the interaction and either engaged in actions themselves or instructed the child to engage in specific actions to build particular circuits. Finally, some dyads were *jointly-directed*; caregivers let children set goals but facilitated children's exploration by asking questions and making suggestions to help children accomplish their goals. We present more details on this coding scheme in the Supplementary Material section.

Second, independent of the caregiver-child interaction style code, we also coded the number of *goal statements* generated by caregivers and children. Goal statements were utterances made by the caregiver or child that stated they had a desire or was working toward a desired outcome regarding the circuits. These statements were marked by the presence of particular verb phrases that directed actions toward the circuits: *going to, want to, trying to, need to, have to, got to* (or *gotta do*), *will do, let's* or the question “What if we <verb denoting action on the circuit blocks>?” Imperatives (“Make the light turn on.” or “Now try it.”) were not considered goal statements, nor were utterances that contained a goal unrelated to the circuits (e.g, “I want to go play with the water now.” “Let's go get a snack.”).

Two undergraduate coders, both blind to the hypotheses of the study, coded 20% of the videos for the caregiver-child interaction style code. Agreement was 81% (Kappa = 0.70). Disagreements were resolved through discussion with one of the authors. The remaining videos were then coded by one of these two undergraduate coders individually. Two different undergraduate coders, also blind to the hypotheses of the study, coded a different 20% of the videos for the goal statements. Agreement was 98% (Kappa = 0.88). The remaining videos were then coded by one of these two undergraduates individually.

#### Actions When Completing a Circuit

Following Sobel et al. (2020), we coded families' play with the exhibit based on whether they constructed each of eight different commonly-constructed circuits. We initially coded videos of dyads' interactions to determine whether groups built any of these circuits, and if so, we noted the time stamp when they completed the circuit in order to demarcate these events for further analysis. Two undergraduate research assistants coded 20% of the data. Agreement was 91% (Kappa = 0.81). Disagreements were resolved by one of the authors. The two undergraduates then proceeded to code the rest of the data independently.

We then counted the number of actions (connecting or disconnecting an alligator clip to a circuit block, or pressing a button) that both the caregiver and the child engaged in during the 30 s before and the 30 s after those circuits were completed. Actions in the 30 s prior to completing the circuit provided a measure of how active caregivers and children were in completing the circuits, and actions in the 30 s immediately afterward served as a control measure for how active caregivers and children were more generally, as these actions did not lead to the completion of circuits in a predictable way. The same 20% of the data were coded by one of the undergraduate research assistants who had coded whether groups completed the circuits, as well as a third undergraduate who had not yet viewed the videos, and both were blind to the hypotheses of the experiment. Agreement (which included all cases where the count was equal or off by 1 action) was 94% (Kappa = 0.91). Disagreements (including all cases where the count was off by 1) were resolved through discussion with one of the authors. These two undergraduates then coded the rest of the data independently.

All other coding is described in the Supplementary Material section, and did not relate to goal setting or completion codes described below.

#### Analysis Strategy

Our analyses focused on the following two questions. First, are there differences between the conditions in caregiver-child interaction style (i.e., whether caregivers, children, or both took the lead in their exploration), in how caregivers or children set goals while playing with the exhibit, or in the actions they each took to complete circuits? Second, does caregiver-child interaction style relate to the actions that caregivers and/or children took to complete the circuits?

Our analyses concentrated on goal setting and actions used to complete a goal because Sobel et al. (2020) found that these two behaviors—as opposed to many others—were directly related to children's engagement with and performance on challenges related to the circuit exhibit. These behaviors also provide two separate pieces of evidence about how involved caregivers were in children's exploration—in directing the goals of what they do with the exhibit, and/or being physically involved in constructing circuits as they played. Other analyses, including analysis of responses to the Attitudes toward Science questionnaire, and other language analyses, are presented in the Supplementary Material section. We did not find significant relations between demographic variables and our measures of interest; these analyses are also reported in the Supplementary Material section.

## Results

**Figure 3:**
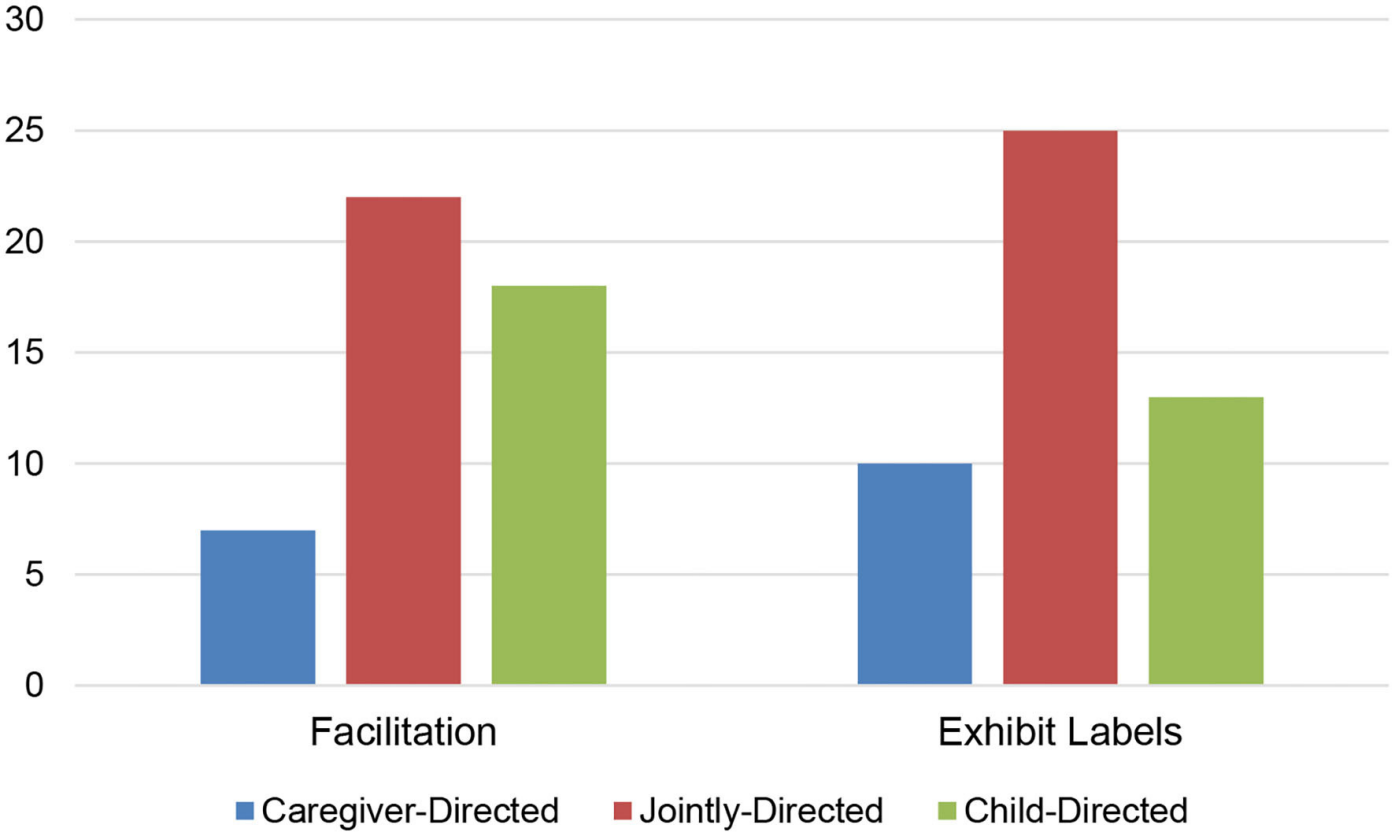
Number of dyads with each caregiver-child interaction style across the two conditions.

We first examined whether there were differences in the way in which caregivers and children played together at the exhibit based on the condition that they were in (facilitation vs. exhibit labels). Our measure of caregiver-child interaction focused on the how goals were set and accomplished while groups explored the exhibit. The distribution of caregiver-child interaction styles (parent-directed, jointly-directed, and child-directed) between the two conditions is shown in Figure 3. There was no difference in the distribution of caregiver-child interaction styles between the facilitation and exhibit label conditions, χ^2^(1, *N* = 95) = 1.52, *p* = 0.47.

We next looked at the proportion of caregivers' and children's utterances that were classified as goal statements. On average, caregivers in the exhibit label condition generated 5.33 goal statements (SD = 6.97) and 7.40 (SD = 8.80) in the facilitation condition. Children generated an average of 2.90 (SD = 3.28) in the exhibit label condition and 4.66 (SD = 4.99) in the facilitation condition. We built Generalized Linear Models, treating the dependent measure as an ordinal response, on both the number of goal statements generated by caregivers and by children, with condition and children's age (in months) as independent variables. Caregivers' goal statements did not differ between the conditions, B = −2.02, SE = 1.58, 95%CI [−5.10, 1.07], Wald χ^2^(1) = 1.64, *p* = 0.20, but did differ with children's age with caregivers of younger children generating more goal statements, B = −0.12, SE = 0.06, 95% CI [−0.22, −0.01], Wald χ^2^(1) = 4.29, *p* = 0.04. Children's goal statements did not differ with age, B = 0.02, SE = 0.03, 95%CI [−0.04, 0.08], Wald χ^2^(1) = 0.45, *p* = 0.50, but did differ between the condition, with children generating more goal statements in the facilitation condition than the exhibit label condition, B = −1.77, SE = 0.85, 95%CI [−3.45, −0.10], Wald χ^2^(1) = 4.32, *p* = 0.04.

We looked at children's actions while groups played with the exhibit. Dyads built more of the eight pre-defined circuits in the facilitation condition (M = 5.13, SD = 1.44) than in the exhibit label condition (M = 3.90, SD = 2.24), Mann-Whitney *U* = 743.50, *z* = −2.90, *p* = 0.004, *r* = 0.30. To isolate the unique variance of condition, we ran a Generalized Linear Model on the number of circuits built, treating the dependent measure as an ordinal response, looking at age (in months), condition (facilitation vs. exhibit labels) and caregiver-child interaction style as independent measures. The overall model was significant, Likelihood Ratio χ^2^(4) = 24.00, *p* < 0.001. There were significant effects of condition B = −1.20, SE = 0.38. Wald χ^2^(1) = 9.79, *p* = 0.002, and of age, B = 0.05, SE = 0.01. Wald χ^2^(1) = 11.99, *p* = 0.001. The main effect of caregiver-child interaction style was not significant.

We next documented whether there were differences in children's and caregivers' actions while playing at the exhibit, and in particular, if there was a difference in how active they each were leading up to connecting a circuit. For each completed circuit, we counted the number of actions performed by the child and by the adult in the 30 s before and the 30 s after completion of the circuit. This allowed us to contrast how each member of the dyad acted while working toward a goal, and after that goal was completed. These data are shown in Tables 3, 4.

**Table 3:** Average number of actions generated by caregivers and children in the 30 s before circuits were completed.

		**Caregiver-child interaction style**		
**Actions by**	**Condition**	**Caregiver-directed**	**Jointly-directed**	**Child-directed**
Caregivers	Exhibit labels	4.69 (2.41)	2.47 (2.36)	0.80 (1.62)
	Facilitation	2.50 (2.08)	0.81 (0.86)	0.56 (0.91)
Children	Exhibit labels	2.58 (1.58)	3.30 (1.96)	3.82 (2.79)
	Facilitation	3.05 (1.57)	3.87 (2.29)	3.65 (1.91)

**Table 4:** Average number of actions generated by caregivers and children in the 30 s after circuits were completed.

		**Caregiver-child interaction style**		
**Actions by**	**Condition**	**Caregiver-directed**	**Jointly-directed**	**Child-directed**
Caregivers	Exhibit labels	3.66 (2.63)	1.70 (2.20)	0.42 (1.06)
	Facilitation	2.10 (2.02)	0.61 (0.64)	0.20 (0.39)
Children	Exhibit labels	3.64 (2.16)	3.72 (2.32)	3.01 (2.02)
	Facilitation	2.45 (1.60)	3.52 (2.23)	4.01 (2.12)

We constructed Generalized Linear Models assuming an ordinal response on the number of actions performed by the adult or child. We considered condition, caregiver-child interaction style, and children's age as independent variables. In each case, factorial models resulted in no significant interactions, and the fit of the model (as measured by BIC) was poorer than a main effect model, so we report only main effect models.

Looking at adults' actions before completion of the circuit, the overall model was significant, Likelihood Ratio χ^2^(4) = 41.83, *p* < 0.001. There was a main effect of condition, with adults generating more actions in the exhibit label condition than the facilitation condition overall, B = 1.15, SE = 0.40, Wald χ^2^(1) = 8.47, *p* = 0.004. There was also a main effect of caregiver-child interaction style, Wald χ^2^(2) = 27.61, *p* < 0.001; adults in caregiver-directed and jointly-directed groups generated more actions than adults in child-directed groups, B = 3.38 and 1.66, SE = 0.64 and 0.49, Wald χ^2^(1) = 27.58 and 11.32, both *p* ≤ 0.001. Moreover, adults in caregiver-directed groups generated more actions than those in joint-directed groups, B = 1.72, SE = 0.52, Wald χ^2^(1) = 10.96, *p* = 0.001. There was not a significant effect of children's age. Looking at adults' actions after circuits were completed, the overall model was significant, Likelihood Ratio χ^2^(4) = 42.85, *p* < 0.001. This result was characterized only by a main effect of caregiver-child interaction style, Wald χ^2^(2) = 31.28, *p* < 0.001. Again, adults in the caregiver-directed groups generated more actions than adults in the other two groups, and adults in jointly-directed groups generated more actions than adults in child-directed groups, all B-values > 1.75, all Wald χ^2^(1)-values > 11.01, all *p* ≤ 0.001. Condition was a marginally significant trend, B = 0.78, SE = 0.41, Wald χ^2^(1) = 3.72, *p* = 0.06. Age was not significant.

Looking at children's actions before the completion of the circuit, the overall model was significant, Likelihood Ratio χ^2^(4) = 9.97, *p* = 0.04, with children's age as the only significant unique predictor of variance, B = 0.04, SE = 0.01, Wald χ^2^(1) = 6.50, *p* = 0.01. Neither condition nor caregiver-child interaction style was significant in this model. Looking at children's actions after the completion of the circuit, the overall model was not significant, Likelihood Ratio χ^2^(4) = 2.31, *p* = 0.68, and none of the three variables was significant on their own.

Finally, we looked at whether various aspects of the demographic information about the sample related to goal setting or the amount of actions caregivers or children generated before or after they completed a circuit. Most of these findings are non-significant. They are detailed in the Supplementary Material section.

## Discussion

This study examined caregiver-child interactions at a circuit block exhibit as family groups played together, when prompts were offered either by a facilitator or by written labels on the exhibit components themselves. We investigated whether either condition would affect dyads' overall interaction style—defined based on whether caregivers or children set goals for what they would do with the circuit blocks—and/or the actions they each took to physically connect the circuits. This study builds on prior work showing that when caregivers were more directive in setting goals for the play, children showed less engagement on follow-up circuit-building challenges, and when caregivers were more active in connecting the circuits, children were subsequently less able to reconstruct circuits on their own (Sobel et al., 2020). In the current study, we focused on how facilitated vs. written prompts offered during the exhibit experience would affect caregiver-child interactions. We used a facilitation style similar to practices commonly used in children's museums and science centers, in which facilitators offered open-ended prompts and questions as families explored the exhibit, and used the same prompts in the exhibit label condition.

Receiving prompts from a facilitator or from exhibit labels did not affect whether caregivers, children, or both collaboratively set goals as they played, and other language measures also did not differ across conditions (see Supplementary Material for analyses). However, when prompts were given by a facilitator, caregivers engaged in fewer actions to connect the circuits. These findings add nuance to previous studies of museum facilitation, which have found that in some cases, facilitators' presence can disrupt or reduce caregiver-child interactions (Pattison and Dierking, 2012, Pattison et al., 2018). Our findings suggest that the presence of facilitators did not shift families' overall interaction style, compared to the presence of written prompts on exhibit labels, but it did shift how much caregivers physically interacted with the exhibit—caregivers were more “hands-off” in exploring the exhibit when facilitators offered prompts than when families read the prompts on the exhibit itself. This condition difference could indicate that facilitators' presence suggested to caregivers that they should limit their own interaction with the exhibit, and instead allow children to take the lead in using the exhibit materials.

We also found that compared to the exhibit label condition, children in the facilitation condition made more goal statements, and dyads completed more circuits while exploring the exhibit. It is possible that because the facilitator was able to observe families' interactions and interject with prompts that were timed at opportune moments when dyads paused during their play, and chosen based on what they had recently done with the circuit blocks (providing more or less challenging prompts from the set of eight, depending on what had happened before they paused), that this may have extended or deepened some aspects of their exploration. It is also possible that both children and caregivers interpreted prompts on the part of the facilitator as pedagogical instruction to continue playing at the exhibit or to build more and more varied types of circuits. In contrast, although families in the exhibit label condition had access to the same prompts, they were not offered at strategic times or in response to aspects of families' play. Therefore, even though facilitation in this study was heavily scripted, the responsiveness of the facilitator may have played a role in the condition effects that we observed.

Our results suggest that the choices museums make about how to convey information can affect the ways that caregivers are involved in exploring exhibits with their children. These findings have implications for children's engagement and learning with this exhibit. Sobel et al. (2020) found that when caregivers were directive, children were less engaged in a subsequent problem-solving task with these exhibit materials, and when caregivers made more actions to complete circuits while they play with their children, children were subsequently less able to construct circuits on their own. In the present study, the exhibit labels we tested had an impact only on caregivers' actions, and not on the frequency of directive interaction styles. Together, the two studies suggest that choosing facilitation over exhibit labels may support children's performance in such problem-solving tasks, in line with prior work showing the importance of children's own actions in supporting their understanding of causal systems (Kushnir and Gopnik, 2005; Sobel and Sommerville, 2010) and their exploration of novel objects (Bonawitz et al., 2011). In contrast, children's engagement in attempting to solve problems may be more influenced by caregivers' involvement than by the manner in which prompts are given. Although we did not conduct follow-up measures in this study, this hypothesis could be tested in future studies.

On the other hand, the presence of written prompts on exhibit labels seemed to support caregivers in being more physically involved in exploring the exhibit and actively making discoveries with their children. There may be situations when the benefits of this type of shared exploration may outweigh the benefits of allowing children to engage in more actions on their own. In particular, for cultures in which children are expected to learn through observation, this way of interacting may feel more natural and in alignment with families' interactions in other settings (Rogoff et al., 2016). In addition, because studies have found that caregivers support children's learning in a wide variety of ways (Swartz and Crowley, 2004; Gaskins, 2008; Beaumont, 2010; Downey et al., 2010), museums may wish to provide multiple avenues for caregivers to be involved—by setting goals together, by physically exploring together, or both. In these cases, exhibit labels may open up more possibilities for caregivers' participation.

Nevertheless, these two strategies are obviously not mutually exclusive and rarely exist separately in real-world museum settings. One limitation of the current study is that the facilitator offered prompts throughout families' entire interaction with the exhibit, rather than “fading” (offering initial support to families and then letting families continue on their own), a more common approach in many museums. Combining more minimal facilitation with exhibit labels might allow museums to blend the benefits of both approaches and also be more responsive to families' needs and ways of learning. With this in mind, our findings can inform facilitation strategies used in museums by highlighting the potential impact of facilitation on caregivers' involvement, helping facilitators notice aspects of families' interactions when deciding how best to support children and caregivers' exploration of museum exhibits.

A second limitation is that this study focused on only one type of exhibit (a hands-on exhibit with connections to STEM learning). Whether the findings would generalize to exhibits that focus on other types of learning or other forms of play remains an open question. Certain aspects of our coding, however, such as the parent-child interaction style coding and the language analysis that we report in the Supplementary Material, have been applied to gear exhibits in multiple museums, as well as other toys with causal properties (Willard et al., 2019; Callanan et al., 2020; Medina and Sobel, 2020), suggesting that similar patterns of interactions are apparent in a range of informal learning contexts. In addition, the specific prompts used in the current study are relevant to exhibit experiences that emphasize hands-on inquiry and open-ended exploration, which are increasingly common in many museum settings (Humphrey and Gutwill, 2017).

Finally, although we did not observe differences in measures of PCI or actions across demographics, the majority of the sample in the current study was highly educated (with 58% of caregivers possessing a BA degree or above) and white (65% of the sample). Additionally, written and verbal prompts were offered only in English, and only one researcher (a white woman) served as a facilitator. Future studies could provide facilitation in other languages or involve a more diverse group of participants and facilitators in order to provide greater opportunities to understand how caregiver-child interactions in informal learning environments might be shaped by families' cultural, socioeconomic, and linguistic backgrounds.

In conclusion, this study brought together prior research on caregiver-child interactions in museum settings and practitioner expertise about the types of exhibit interventions that museums often utilize to support and extend families' interactions and learning. The findings from this line of work deepen our understanding of how museum settings can be designed and facilitated to provide more engaging learning experiences for children and their families. In addition, the methods we used to contrast commonly-used strategies can inform future studies with a range of settings and audiences.

## Data Availability Statement

The raw data supporting the conclusions of this article will be made available by the authors, without undue reservation.

## Ethics Statement

The studies involving human participants were reviewed and approved by Brown University Institutional Review Board. Written informed consent to participate in this study was provided by the participants' legal guardian/next of kin.

## Author Contributions

SL conducted the data collection and oversaw coding with DS. DS performed the data analysis. SL and DS wrote the initial draft. All authors contributed to editing the manuscript and conceptualized the project together.

## Conflict of Interest

The authors declare that the research was conducted in the absence of any commercial or financial relationships that could be construed as a potential conflict of interest.

## Footnotes

^1^All families provided written consent to participate. As required by our IRB protocol, 7-year-olds (but not younger children) also needed to provide verbal assent to participate.
