# Supplementary material for: Effects of Facilitation vs. Exhibit Labels on Caregiver-Child Interactions at a Museum Exhibit
Source: Front Psychol. 2021 Mar 12;12:637067. doi: 10.3389/fpsyg.2021.637067 (PMC8006285; doi:10.3389/fpsyg.2021.637067)
Supplement: Supplementary file 1 [file Table_1.DOCX]

Effects of facilitation versus exhibit labels on caregiver-child interactions at a museum exhibit

Supplemental Materials

**Caregivers’ Attitudes towards Science**

The Attitudes toward Science survey contains 15 statements on which participants responded on a 7-point scale from 1 (mostly disagree) to 7 (mostly agree). This measure contained statements about one’s personal interest in science (e.g., “I would enjoy being a scientist”), one’s views of science and scientists (e.g., “Scientists are among the smartest people”), and one’s beliefs about the utility of science (e.g., “Thinking like a scientist is only useful when taking a test in a science class”). Caregivers’ overall attitudes toward science scores were calculated based on their mean agreement across the 15 items (reversing scoring the items where appropriate). Ninety-two of the participating 95 caregivers filled out this questionnaire. Their mean score was 5.01 out of a possible 7.

There was no difference between these scores in the Facilitation condition (*Median* = 5.13) and the Exhibit Labels condition (*Median* = 4.73), Mann-Whitney U = 879.50, *z* = -1.40, *p* = .16. Median scores for the three parent-child interaction groups are shown in Table S1. There was a differences among the three groups, Kruskal-Wallis H(2) = 6.52, *p* = .04, η^2^ = .05. Simple effect analysis revealed that the only significant pairwise comparison was that jointly-directed group scored higher than the parent-directed group, *p* = .03 with a Dunn-Bonferroni correction.

Sobel et al. (2020) also administered the Attitude towards Science measure and performed the same parent-child interaction style coding analysis as we did here. Inspection of their data reveals that they did not find a significant difference in Attitudes towards Science scores among the three parent-child interaction styles, Kruskal-Wallis H(2) = 0.91, *p* = .63, η^2^ = -.01^[[1]](#footnote-1)^. Callanan et al. (2020) also used this parent-child interaction scoring system and administered the Attitudes towards Science measure. Inspection of their data also shows that they also did not find a difference among the three parent-child interaction styles, Kruskal-Wallis H(2) = 2.01, *p* = .37, η^2^ = .00. Averaging these effect sizes together reveals a mean η^2^ = .01, which suggests that the difference has a very small effect.

Because there was a relation between parent-child interaction style and attitudes towards science in this sample, we reran all the analyses on caregivers’ and children’s actions before and after they complete circuits during their play, including caregivers’ attitudes towards science as a covariate. In none of those analyses was attitudes towards science a significant predictor of caregivers’ or children’s actions before or after completing a circuit, nor did any of the significance levels reported in the main text change.

Table S1

*Median Scores on Attitudes towards Science, Parent Education Level and Household Income across the three parent-child interaction scores*

|  | Attitudes towards Science Score | Caregivers’ Education Level | Household Income Level |
| --- | --- | --- | --- |
| Parent-Directed | 4.56 (0.76) | High School Diploma | $50-70K |
| Child-Directed | 4.80 (0.66) | BA | $70-90K |
| Jointly-Directed | 5.20 (0.76) | MA | $90-120K |

**Other relations among demographic factors, attitudes towards science, and goal setting and completion**

We ran zero-order correlations among attitudes about science and children’s age, caregivers’ age group, the frequency with which families visited the museum, caregivers’ reported gender, the demographic, family income group and caregivers’ education group. The frequency with which families visited the museum correlated with caregivers’ Attitudes towards Science score, *r_s_*(90) = .27, *p* = .009. Similarly, caregivers’ income correlated with Attitudes towards Science score, *r_s_*(87) = .24, *p* = .02. No other significant correlations were found among these variables.

We also looked at whether these variables differed among the parent-child interaction style groups. Scores on the attitudes towards science question are documented above. There were no differences in children’s age, caregivers’ age, or caregivers’ reported gender. There were differences among the groups regarding both household income level and parent’s education level, Kruskal-Wallis H(2) = 9.35 and 15.11, *p* = .009 and .001, η^2^ = .08 and .14 respectively. These data are shown in Table S2. As with the Attitudes towards Science scores, we were able to compare these results to the sample collected by Sobel et al. (2020), who did not find these relations, Kruskal-Wallis H(2) = 1.60 and 2.70, *p* = ..45 and .25, η^2^ = -.004 and .007 respectively. Averaging these together, these findings suggest small to moderate effects, η^2^ = .04 for household income, and η^2^ = .07 for caregivers’ education level.

Because caregivers’ education level and household income were related to parent-child interaction style in this sample, we reran the ordinal analysis reported in the main text on caregivers’ and children’s actions before and after completion of the circuit, adding caregivers’ education level and household income as correlates. In none of those analyses did any of the significance levels reported in the main text change. Caregivers’ actions both before completion of a circuit, B = -0.35, SE = 0.17, Wald χ^2^(1) = 4.37, *p* = .04, and after completion of a circuit, B = -0.40, SE = 0.18, Wald χ^2^(1) = 5.13, *p* = .02, were inversely correlated with education level. In both cases, caregivers with higher levels of education acted less, controlling for all other factors. This finding was unique to this sample. Investigation of the Sobel et al. (in press) data suggests that education level did not predict caregivers’ actions while they used the exhibit.

**Language Analysis**

To analyze the language generated by caregivers and children, families’ conversations while playing with the exhibit were transcribed and then parsed into individual utterances. All utterances were timestamped so that their occurrence could be connected to specific actions being performed. Coding was done through access to the transcript as well as watching the video. Caregivers’ utterances were coded if they were directed specifically at the participating child. Children’s utterances were coded if they were directed specifically at the participating parent. Nonverbal behavior, including nods, shrugs, head shakes, laughs, or gasps was indicated in the transcript, but only coded when relevant to one of the verbal coding categories described below. When a parent or child made a false start in their utterance (e.g., “I wonder if—let’s put it over here”), the code was based on only the second part of the sentence, ignoring the false start. The context of the video determined which code was assigned.

We analyzed the transcripts of dyad’s interactions with the exhibit for the language generated by caregivers and children. The coding scheme we used was similar to the one used in Callanan et al., (2020) and Sobel et al. (2020). All utterances on the part of the parent and child were coded according to the following coding scheme. For simplicity and to connect to how we analyzed these data, we organized this scheme into a hierarchy. This hierarchy was given to our coders, so that they could more easily categorize certain utterances into one part of the coding system, and then determine the precise code. This hierarchy and codes are both shown in Table S2.

Table S2

*Categories and Examples of Language Coding*

| Major Category 1: Explaining | |
| --- | --- |
| Category | Definition and examples |
| Causal Connections | Making a statement about how an action leads to a consequence (e.g., “when you connect it, it makes this motor spin”, “pressing the button turns it off”, “the battery makes it go”). These statements should include mention of both the cause and the effect in the exhibit (what happened, and what made it happen), and *may* include the word “because” (e.g., “the light turned on because both sides are connected”). |
| Causal Connections Question | Asking about what could cause a given effect or why something happened (e.g., “why isn’t it working?” “how did that happen?” “how can we make the light turn on?”) These questions usually included the words “why” or “how.” |
| Making Predictions | Suggesting what *will happen* as a consequence of some action. (For example, “I think it will work if we add another battery..”) |
| Making Predictions Question | Asking for predictions about the effect or outcome of an action (e.g., “what do you think will happen if we connect all of them?” “what happens if you turn it the other way?”) |
| Personal Connection/Prior Experience | Statement that relates the experience to some previous personal experience/memory or a piece of information with personal relevance (e.g., “this is like the Snap Circuits you have at home,” “this is how the light switch in your room works”). |
| Personal Connection Question | Question that requests such a personal connection (e.g., “what does this remind you of that we did last summer?”; “What do we have in our living room that has a battery?” ) |
| Scientific Principle | Statements that are meant to communicate a general science concept or generalizable knowledge related to circuits/electricity (e.g., “batteries provide power”, “electricity goes through wires” “circuits have to be closed”); can include analogies if stated generally (“this is like how light switches work”, “a circuit is like a circle”).  Statements that include a science word, but that only apply to what families are doing in that moment, and not electricity/circuits in general were not coded in this manner (e.g., “The wire is connected to the battery.” would be coded as Labeling or Describing (see below). Similarly, labeling parts of the display (e.g,. “That’s a battery”) were not given this code. |
| Scientific Principle Question | Question asking for general information about electricity/circuits (e.g., “What do batteries do?” “Do you know what a circuit is?”) |
| Major Category 2: Talk about Action or the Exhibit | |
| Labeling or Describing | Naming parts of the exhibit or talking about properties of the exhibit. For example, labeling parts (“A battery.” “that’s a light”), stating whether the circuit (or parts of the circuit) are working (“It’s not going.” “Now it’s working!”), descriptions of how the parts connect (“all of them are connected”, “there are a lot of wires”), descriptions of what pieces do (“it’s lighting up”, “it’s going really fast” “the wires attach to the pegs”). The subject of these statements was almost always an object in the exhibit. |
| Labeling or Describing Question | Asking for a label or description of something in the exhibit. For example, “Is it connected?” “Is the motor spinning?” “Where is the battery?” “how many are there?” |
| Directing another’s Action | Imperative statements telling the other person what to do (but with no causal link mentioned). Directions or instructions about what to do. For example, “Connect that one over there.” “Move that one over.” “Use this battery.” “Try it.” This category also included statements about what someone needs to do, should do, has to do For example, “You need to connect it.”; “You should try adding another battery.”; “You have to clip it on both sides.” This code was also used for statements of this form phrased with “we” as the subject. (“We need to __” “we have to ___”) |
| Suggesting/Scaffolding Action | Prompting or suggesting an action, not as an imperative statement but in a more subtle form or as a possibility rather than as a direct instruction or imperatives. For example, “Maybe there’s another way to do it.” “What if you use the light instead?” “You can use more wires.”). This code includes asking or requesting that someone perform an action (but with no causal link mentioned) or asking to do something together. For example, “Can you connect it?” “Can you press the button?” “Do you want to ___?” “Want to try ___?” “Can we try ___?” “What if we ___” |
| Narrating Own or Other’s Action | Statements describing what one or someone else did, is doing, or will do (without directing or making a causal connection to the exhibit). The subjects of these sentences were always a person. For example, “I’m going to clip this one to that one.” “I’ll do it.” “You connected them all!” This code was also used for statements that met this criteria with a plural subject (e.g., “We added the button.”), and fragments of sentences that accompany an action (e.g., “and there!” said while connecting a wire). These utterances could be in question form if someone is asking for clarification about what is happening or what someone is doing (e.g., “Can I connect it?”, “Did you move it?”) and is not requesting that the other person do something (which would be coded as Suggesting/Scaffolding Action above). |
| Open Ended Questions | Asking questions that do not include a specific suggestion – i.e., the answer is not constrained in any way. For example, asking someone about what they want to do without using a leading or closed-ended question. For example: “What next?” “Now what?” “What do you want to do?”; “Which one are you going to try?” “Where are you going to put that one?” Also includes asking about someone else’s preferences (e.g., “Do you want me to help?” “Want to keep playing?”) or opinions/ideas (“What do you think?”) |
| Major Category 3: Other Kinds of Talk | |
| Guiding Attention | Suggesting that the other person focus on some part of the exhibit (but without describing). For example, “Look over there and see what’s happening!” “Watch this Mom!” Also includes saying someone’s name to get their attention |
| Guiding Attention Question | Utterance that asks for attention. For example, “Can you see how it looks from up above?” |
| Emotion | Expressions of emotions such as awe, frustration, pride, humor. For example, “Wow! Cool!” as well as “Uh oh!” |
| Emotion Question | A question that asks about an emotional reaction. For example, “Did that surprise you?” |
| Praise | Utterance that praises another’s action. For example, “Good job!” or “You’re so smart!” |
| Praise Question | Utterance that request praise from another person. For example, “Did I do a good job?” |
| Other On-Task Utterance | Any utterance that don’t fit into categories above (or don’t have enough information to categorize) but are on-task because they are focused on the exhibit. For example, “Yes”, “Hmm”, “Okay”, “Maybe”, “You know what?”, etc., when relevant to exhibit. Coders were encouraged only to use this code when there was not enough information from the statement and context to code it in any other category. |
| Other Off-Task Utterance | Any utterance that does not fit into categories above and are generally off task. For example, “I’m hungry” or “Can we leave now?” |

Two undergraduate research assistants each coded a subset of approximately 20% of the data. Agreement on these codes was 94% (Kappa = .91). Disagreements were resolved through discussion with one of the authors. These two research assistants then each coded the remainder of the dataset individually.

Looking across the conditions, language was analyzed as a proportion of the total number of utterances generated by the parent or the child. There was no difference in caregivers’ language across conditions for any of these categories, Mann-Whitney *U-*values between 841.00-983.00, all *z*-values < |1.54|, all *p*-values > .12. The only significant difference in the proportion of children’s language was in the category of *other kinds of talk*, Mann-Whitney *U* = 683.00, *z =* -2.52, *p* = .01, *r* = .26. This difference, however, reflected the fact that children in the exhibit label condition generated a greater proportion of off-topic utterances than children in the facilitation condition (11% vs. 5%), Mann-Whitney *U* = 709.50, *z* = -2.35, *p* = .02, *r* = .24.

1. η^2^ is usually conceptualized as having a minimal value of 0. However, it can be a negative number. Okada (2016) argued that it should be fit into analyses such as these as a negative number, and not as a value of 0. [↑](#footnote-ref-1)
